# Alteration of the Immune Response and the Microbiota of the Skin during a Natural Infection by *Vibrio harveyi* in European Seabass (*Dicentrarchus labrax*)

**DOI:** 10.3390/microorganisms9050964

**Published:** 2021-04-29

**Authors:** María Cámara-Ruiz, Isabel M. Cerezo, Francisco A. Guardiola, José María García-Beltrán, M. Carmen Balebona, Miguel Ángel Moriñigo, María Ángeles Esteban

**Affiliations:** 1 Immunobiology for Aquaculture Group, Department of Cell Biology and Histology, Faculty of Biology, Campus Regional de Excelencia Internacional “Campus Mare Nostrum”, University of Murcia, 30100 Murcia, Spain; Maria.camara1@um.es (M.C.-R.); faguardiola@um.es (F.A.G.); josemaria.garcia4@um.es (J.M.G.-B.); 2 Departamento de Microbiología, Universidad de Málaga, 29071 Málaga, Spain; b22ceori@uma.es (I.M.C.); balebona@uma.es (M.C.B.); morinigo@uma.es (M.Á.M.)

**Keywords:** immune response, skin immunity, microbiota, vibriosis, European sea bass (*Dicentrarchus labrax*)

## Abstract

Disease outbreaks continue to represent one of the main bottlenecks for the sustainable development of the aquaculture industry. In marine aquaculture, many species from the *Vibrio* genus are serious opportunistic pathogens responsible for significant losses to producers. In this study, the effects on the immune response and the skin microbiota of European sea bass (*Dicentrarchus labrax*) were studied after a natural disease outbreak caused by *V. harveyi*. Data obtained from infected and non-infected fish were studied and compared. Regarding the local immune response (skin mucus) a decrease in the protease activity was observed in infected fish. Meanwhile, at a systemic level, a decrease in protease and lysozyme activity was reported while peroxidase activity showed a significant increase in serum from infected fish. A clear dysbiosis was observed in the skin mucus microbiota of infected fish in comparison with non-infected fish. Moreover, *V. harveyi*, was identified as a biomarker for the infected group and *Rubritalea* for healthy fish. This study highlights the importance of characterizing the mucosal surfaces and microbial composition of the skin mucus (as a non-invasive technique) to detect potential disease outbreaks in fish farms.

## 1. Introduction

Due to the intensification of the aquaculture industry, outbreaks of disease are being increasingly reported and represent a challenge to the expansion of aquaculture production causing significant economic losses to the sector [1]. Sometimes, the spread of infectious diseases also negatively affects varied subsectors related to aquaculture [2]. In marine aquaculture in particular, many *Vibrio* spp. are serious opportunistic pathogens, which represent the most prevalent bacterial diseases affecting invertebrate and vertebrate species (e.g., finfish, shellfish, and shrimp) [3,4]. The main changes in behavior in fish suffering vibriosis are lethargy, anorexia or abnormal swimming patterns among other things. Typically, other external changes observed are ulcerative skin lesions, abdominal distension, gill necrosis and darkened skin. *Vibrio* spp. bacteria usually colonize gills, skin, gut, among other internal organs of the infected host [5]. If untreated, the infection may become systemic, leading to the mortality of the host [3].

Fish are in continuous contact with a broad spectrum of microorganisms which inhabit the aquatic environment and, therefore, they have developed multiple mechanisms to be able to fight infections. Innate immunity provides the first line of defense mechanism in fish [6]. In fact, it is considered highly essential because of their less developed acquired immune response [7]. Innate immunity humoral factors include molecules, such as lysozyme and proteases, that are soluble in plasma and skin mucus [8]. These molecules utilize a wide range of proteins and glycoproteins, which are able to destroy or inhibit the growth of potentially infectious microorganisms [9]. The fish immune system includes primary and secondary lymphoid organs. Among the secondary lymphoid organs is the mucosa associated lymphoid tissue (MALT). The mucosal surfaces (gill, skin, gut, and nose) form a thin physical barrier and are essential in the course of infections since the majority of infectious agents initiate their process of infection at such sites [10]. Such mucosal surfaces are coated by a mucus layer, which is continuously being secreted and it is plenty of varied and potent bioactive molecules. The mucus layer interacts directly with the microbial community present at the site [6].

Over the last few years, researchers have studied how the microbiota exerts effects on the fish immune system, which is critically important for fish disease resistance [11]. In fact, it has been suggested that the interaction between the microbiota and the teleost immune system could potentially shift commensals into opportunists or pathogens under different stress/disease scenarios [6]. It is acknowledge that microbiota influences the teleost immune system through microbe-associated molecular patterns (MAMPs), acting locally on the mucosal sites or even systemically if such MAMPs are able to enter host circulation [6]. However, most of the studies focusing on the crosstalk between the microbiota and teleost immune system have focused on the gastrointestinal tract [11,12].

Thus, the aim of the present work is to study the modulation of skin mucosal immune response and the skin microbial composition using 16S rRNA next generation sequencing (NGS) during the course of a natural episode of infection by *V. harveyi* in a very important Mediterranean farmed fish species, European sea bass (*Dicentrarchus labrax*).

## 2. Materials and Methods

### 2.1. Fish Acclimatization and Disease

A total of 60 European sea bass specimens were obtained from a local fish farm, (Alicante, Spain) and transported to the Marine Fish Facilities at the University of Murcia. Fish were randomly distributed in three identical re-circulating seawater aquaria (250 L) (20 fish per tank). With a flow rate of 900 L h^−1^ and a salinity of 28‰, the water temperature was held at 20 ± 2 °C. The photoperiod was set to 12 h of light and 12 h of darkness. A commercial pellet diet (Skretting, D2) was fed to the fish at a rate of 2% body weight per day. The University of Murcia’s Ethical Committee accepted all experimental protocols.

During the acclimatization period, ten days after arrival to the facility, macroscopic skin lesions were observed in some animals from all tanks what made us suspect a possible infection. A total of 18 specimens (average weight 22.15 ± 5.66 g, average length 12.22 ± 0.89 cm) were sampled. Out of the 18 specimens, 9 were selected (3 from each experimental tank) due to the notable macroscopic skin lesions (infected group) while another 9 specimens were selected (3 from each experimental tank) with no external signs of disease (non-infected group). More specifically, 6 co-habitant fish were sampled from each experimental tank (3 infected and 3 non-infected).

### 2.2. Fish Sampling

Prior to sampling, fish were anesthetized using clove oil (0.1 g L^−1^, Guinama^®^). Firstly, in order to demonstrate a possible bacterial etiology of the macroscopic lesions observed and then, of the disease outbreak, sterile cotton swabs were gently rubbed against the macroscopic wounds and spread on plates of *Flexibacter maritimus* medium (FMM, Labconda, Madrid, Spain). Moreover, the mid-kidney (MK) and head-kidney (HK) of fish showing external signs of disease were also sampled in order to demonstrate that the infection was at the systemic level. Plates were incubated at 25 °C up to 48 h.

Subsequently, skin mucus samples were collected using the method described by Guardiola et al. [13]. The skin mucus was obtained by gently rubbing the lateral surfaces of sea bass specimens with a cell scraper, taking care not to contaminate the samples with blood, urogenital, or intestinal excretions. Collected skin mucus samples were vigorously shaken before being centrifuged (2000× *g*, 10 min, 4 °C) and stored at −20 °C before use. Blood samples were collected from the caudal vein with an insulin syringe. The blood samples were allowed to clot at 4 °C for 4 h, centrifuged (10,000× *g*, 5 min, 4 °C) and stored at −20 °C until use. Furthermore, the body weight and length of each fish was measured.

### 2.3. Bacterial Identification

By amplification and sequencing of a fragment of 16S rDNA, bacterial isolates grown on inoculated plates from skin lesions, MK, and HK were classified to the species level. Complete genomic DNA was extracted from bacteria using the Thermo Scientific Gene JET Genomic DNA Purification Kit (Thermo Fisher Scientific, Waltham, MA, USA). Afterwards, this fragment was amplified using the universal primers SD-Bact-0008-a-S20 (5′ AGA GTT TGA TCC TGG CTC AG 3′) and SD-Bact-1492-a-A-19 (5′ GGT TAC CTT GTT ACG ACT T) [14]. Polymerase chain reactions (PCR (were carried out in a 50 μL reaction mixture that included 5 pmol of each primer, 0.2 mM dNTPs mix, 10X DreamTaq Buffer, 2 mM MgCl_2_, 1.25 U DreamTaq DNA Polymerase (Thermo Fisher Scientific, Waltham, MA, USA) and 1 μL of colony DNA (~ 100 ng/ul). The PCR profile was as follows: 2 min at 95 °C, followed by 35 cycles of 30 s at 95 °C, 40 s at 52 °C and 1.3 min at 72 °C and a final step 5 min at 72 °C. The products of the polymerase chain reaction were electrophoresed on a 1% agarose gel and visualized using ultraviolet transillumination. Before sequencing the PCR product, an ExoSAP-IT purification step was performed. For this, 9 µL of each PCR product were mixed with 0.5 µL of ExoI (20 U µL^−1^) (Thermo Fisher Scientific, Germany). The PCR products were enzymatically purified (37 °C for 15 min, 80 °C for 15 min). Subsequently, they were then sequenced using Sanger technology at Macrogen Spain (Madrid, Spain). Using the Basic Local Alignment Search Tool (BLAST) software, the sequences were then compared to those in the GenBank databases (www.ncbi.nlm.nih.gov/blast, accessed on 27 April 2021).

### 2.4. Immune Parameters

#### 2.4.1. Total Protein Levels

The protein concentration in skin mucus and serum samples was determined using the method described by Ross et al. [15]). Serial dilutions of bovine serum albumin (Sigma-Aldrich, St. Louis, MO, USA) were used as a standard. Plates were read at 550 nm in a plate reader (SPECTROstar^nano^, BMG Labtech, Ortenberg, Germany). The total protein concentration present in each sample was expressed as mg mL^−1^.

#### 2.4.2. Total Immunoglobulins

Total immunoglobulins in skin mucus and serum samples were determined using Bradford reagent. Briefly, 50 μL of the samples were mixed with 50 μL of polietilenglicol (12%) and incubated for 2 h at room temperature (RT). As standard, the same volumes of bovine serum albumin (Sigma-Aldrich) were used in serial dilutions. After the incubation time, samples were centrifuged (2000× *g*, 10 min, RT). Then, 5 μL of the supernatant were collected and placed in a 96-well flat-bottomed together with 250 μL of the Bradford reagent (Sigma-Aldrich). After incubating the plates (10 min, RT, in darkness), the absorbance was read at 550 nm in a plate reader (SPECTROstar^nano^, BMG Labtech, Ortenberg, Germany). Total immunoglobulins were calculated by subtracting the value obtained to the total protein levels previously obtained. Total immunoglobulins present in each sample were expressed as mg mL^−1^.

#### 2.4.3. Peroxidase Activity

The peroxidase activity in skin mucus and serum samples was measured with the same method as described by Quade and Roth [16], with slight modifications. Samples without skin mucus or serum were used as blanks. Plates were read at 450 nm in a plate reader (SPECTROstar^nano^, BMG Labtech, Ortenberg, Germany). Peroxidase activity present in each sample was expressed as units mL^−1^.

#### 2.4.4. Total Immunoglobulin M Levels

Total immunoglobulin M (IgM) levels were analyzed using the enzyme-linked immunosorbent assay (ELISA) [17]. Negative control consisted of samples without skin mucus, serum or primary antibody. Plates were read at 450 nm in a plate reader (SPECTROstar^nano^, BMG Labtech, Ortenberg, Germany). Data are presented as units mL^−1^ for each sample value.

#### 2.4.5. Protease Activity

Protease activity was measured using the azocasein hydrolysis assay with slight modifications [15]. Skin mucus or serum were replaced by trypsin (5 mg mL^−1^, Sigma Aldrich) for the positive controls or by ammonium bicarbonate buffer for the negative controls. Plates were read at 450 nm in a plate reader (SPECTROstar^nano^, BMG Labtech, Ortenberg, Germany). Activity for each sample was expressed as % protease activity in relation to the controls.

#### 2.4.6. Antiprotease Activity

Antiprotease activity of skin mucus or serum was determined by the ability of skin mucus or serum to inhibit trypsin activity [18]. Skin mucus or serum were replaced by ammonium bicarbonate and trypsin (5 mg mL^−1^, Sigma Aldrich) for the positive controls or by ammonium bicarbonate buffer for the negative controls. Plates were read at 450 nm in a plate reader (SPECTROstar^nano^, BMG Labtech, Ortenberg, Germany). Activity for each sample was expressed as % antiprotease activity in relation to the controls. Antiprotease activity was not detected in skin mucus.

#### 2.4.7. Lysozyme Activity

Lysozyme activity was measured according to a turbidimetric method with slight modifications [19]. Lysozyme activity for each sample was expressed as µg mL^−1^ of hen egg white lysozyme eq. activity.

### 2.5. Skin Mucus Microbiota Analysis

#### 2.5.1. Sequencing Preparation and Library Generation

Total DNA of all samples was extracted from skin mucus using protocol by Martinez et al. [20], with minor modifications. DNA integrity and purity were monitored on 1% agarose gels. Concentration was determined by using Qubit 2.0 fluorimeter (Thermo Fisher Scientific, Waltham, MA, USA). 8 samples of non-infected fish and 7 samples of infected fish passed DNA quality requirements for library preparation and sequencing. Amplification of 16S rDNA gene (V3-V4 region) was carried out using the primers 341F (5′-CCTAYGGGRBGCASCAG-3′) and 806R (5′-GGACTACNNGGGTATCTAAT-3′). All PCR reactions were carried out with Phusion^®^ High-Fidelity PCR Master Mix (New England Biolabs, Ipswich, MA, USA). Amplicons were purified with Qiagen Gel Extraction Kit (Qiagen, Hilden, Germany), quantified via Qubit and mixed at equal density ratios. The libraries, generated with NEBNext^®^ UltraTM DNA Library Prep Kit for Illumina, were sequenced on an Illumina Hiseq Platform (2 × 250 pb) (Novogene, Cambridge, UK).

#### 2.5.2. Sequencing Data Processing

Paired-end reads were assigned to samples based on their unique barcodes and truncated by cutting off the barcode and primer sequences. Paired-end reads were merged by using FLASH (V1.2.7) [21]. Then, the assembled reads were quality-filtered, with Phred quality score average ≥20, according to the QIIME (V1.7.0) [22] quality-controlled process. UCHIME [23] was used to detect and remove chimera sequences.

#### 2.5.3. Operational Taxonomic Units (OTU) Cluster and Taxonomic Annotation

The remaining representative, non-chimeric sequences were then analyzed with UPARSE software (V7.0.1001) and assigned into operational taxonomic units at 97% similarity cut-off. Annotation at each taxonomic rank was performed against the SILVA Database (138 release) with Mothur software (confidence threshold 0.8–1).

After generating the taxonomic profile of samples, rarefaction curves were calculated. OTU abundance information was normalized using the sequence number corresponding to the sample with the least number of reads for which the rarefaction curves were asymptotic (47,067). Subsequent analysis of alpha diversity (Chao1, Shannon, inverse Simpson indices) and beta diversity was performed using this output normalized dataset.

#### 2.5.4. Alpha and Beta Diversity

Alpha diversity was estimated based in Chao1, Shannon and inverse-Simpson indices. Rarefaction curves were obtained by plotting the number of observed OTUs against the number of sequences and a good coverage coefficient was calculated in order to determine the level of sequencing depth. All these calculations were performed with QIIME (V. 1.7.0) and displayed with R software (V. 2.15.3).

Beta diversity analysis was used to evaluate differences of samples in species complexity. Non-metric multidimensional scaling (NMDS) analysis was performed by using R software (V. 2.15.3). Linear discriminant analysis (LDA) effect size (LEfSe) analysis was conducted to determine microbial biomarkers of each mucus microbiota.

### 2.6. Statistical Analysis

Results related to the immune parameters were expressed as means ± standard error of mean (SEM). The normality of the variables was confirmed by the Shapiro–Wilk test while the homogeneity of variance was confirmed by the Levene test. Data were statistically analyzed by Student’s *t*-test to determine significant differences between experimental groups (infected and non-infected). The significance level was 95% in all cases (*p* < 0.05). Statistical analyses were conducted using GraphPad Prism 8 and differences were considered when *p* < 0.05. All the determinations were performed in triplicates.

Differences in alpha diversity indices between groups were determined with a *t*-test performed with R software. NMDS was carried out in R (V. 2.15.3.) and the Anosim test was used to evaluate the significance of variations between groups (Vegan package: anosim function). LEfSe analysis was performed using LEfSe software [24] with an alpha value of 0.05 for both the factorial Kruskal–Wallis rank sum test and pairwise Wilcoxon test and a threshold of 2.0 for the LDA. In all the cases, differences were considered significant for *p* < 0.05.

## 3. Results

### 3.1. Skin Lesions and Bacterial Characterization

**Figure 1:**
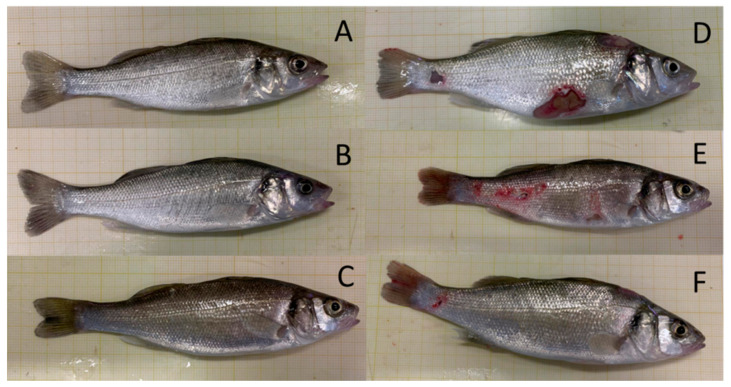
Macroscopic photographs of representative European sea bass specimens belonging to the non-infected (**A**–**C**) and infected group (**D**–**F**).

As mentioned earlier, macroscopic skin lesions were observed in some animals from all tanks, while others had a normal appearance (Figure 1). Bacterial strains isolated from the skin wounds, MK and HK samples of injured European sea bass were identified as *V. harveyi* based on 16S rRNA analysis and grown as pure culture in 73.40% of the samples (Table 1). The presence of bacterial growth from MK and HK samples and ulcers indicated the infection by vibriosis of the animals with symptoms, so the fish were divided into infected and non-infected groups for further analysis.

### 3.2. Immune Response

#### 3.2.1. Skin Mucus Immunity

**Figure 2:**
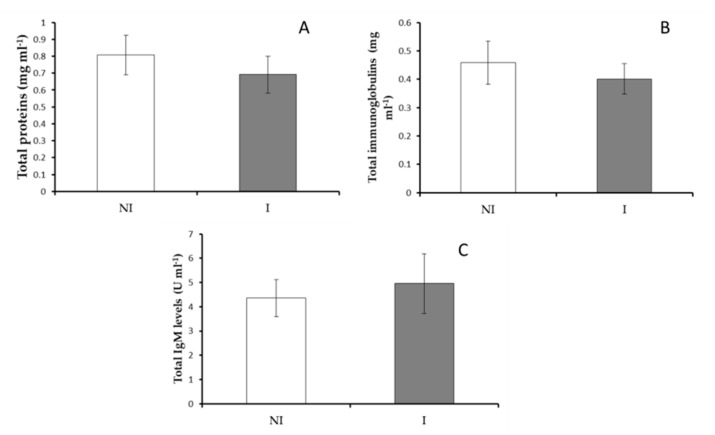
Skin mucus immune response in non-infected (NI) and infected (I) European sea bass. (**A**) Total protein levels, (**B**) total immunoglobulin levels and (**C**) total IgM levels. Data are represented as means ± standard error of the mean (SEM, *n* = 9).

**Figure 3:**
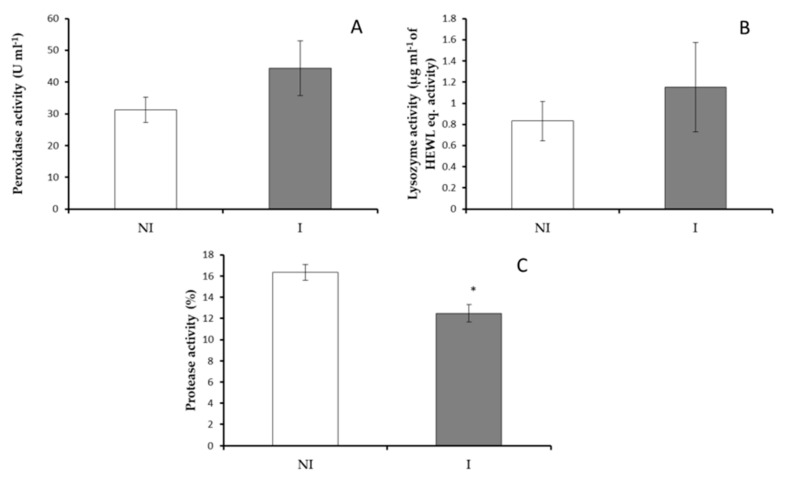
Skin mucus immune response in non-infected (NI) and infected (I) European sea bass. (**A**) peroxidase, (**B**) lysozyme, (**C**) protease activities. Data are represented as means ± SEM (n = 9). Asterisks denote significant differences between groups (*p* < 0.05).

The values of total protein, total immunoglobulins, total IgM levels in skin mucus of infected and non-infected fish did not show significant variations (*p* > 0.05) (Figure 2). However, a significant decrease (*p* < 0.05) in skin mucus protease activity was observed in infected fish compared to non-infected fish (Figure 3A) whilst peroxidase and lysozyme activities did not show any significant variations (Figure 3B,C). Finally, antiprotease activity was undetected in European sea bass skin mucus samples.

#### 3.2.2. Serum Immunity

**Figure 4:**
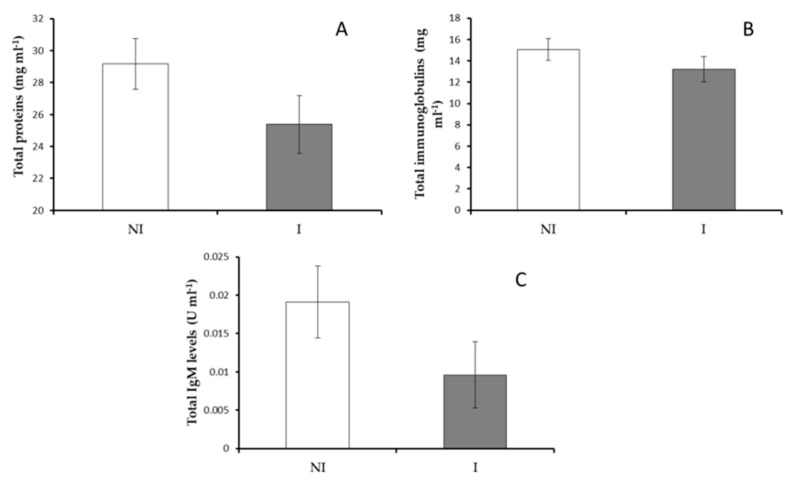
Serum immune response in non-infected (NI) and infected (I) European sea bass. (**A**) Total protein levels, (**B**) total immunoglobulin levels and (**C**) total IgM levels. Data are represented as means ± SEM (n = 9).

**Figure 5:**
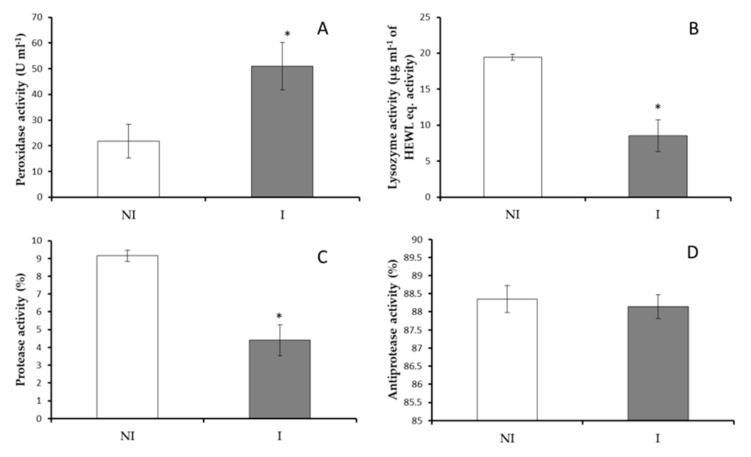
Serum immune response in non-infected (NI) and infected (I) European sea bass. (**A**) peroxidase, (**B**) lysozyme, (**C**) protease and (**D**) antiprotease activities. Data are represented as means ± SEM (n = 9). Asterisks denote significant differences between groups (*p* < 0.05).

No significant differences were observed in the total protein, total immunoglobulins and total IgM levels in serum from fish non-infected or infected with *V. harveyi* (Figure 4). However, a significant increase (*p* < 0.05) in peroxidase activity was observed in infected fish compared to results obtained in the non-infected fish (Figure 5A). Contrarily, the values of lysozyme and protease activities were significantly decreased in serum of fish infected regarding non-infected ones (Figure 5B,C). No significant differences were observed in serum antiprotease activity between infected and non-infected fish groups (Figure 5D).

### 3.3. Skin Microbiota Diversity and Composition

**Figure 6:**
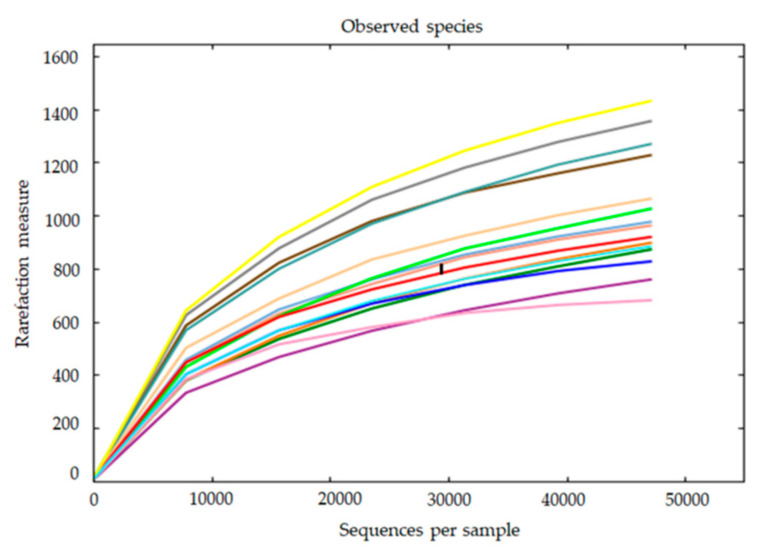
Rarefaction curves obtained from sequencing data of the different samples included in this study.

A total of 1,743,391 high quality reads, corresponding to 116,226 ± 6,283.30 reads per sample was obtained. Regarding experimental groups, 100,776 ± 7,807.40 mean reads corresponded to the non-infected group (NI), 133,884 ± 4,323.35 mean reads to the infected group (I), and a minimum of 60,282 reads and a maximum of 148,579 reads. A total of 3555 OTUs were assigned at 97% identity threshold. Rarefaction curves approximated saturation with 47,067 reads. Thus, this number of sequences was considered appropriate for normalization (Figure 6).

**Figure 7:**
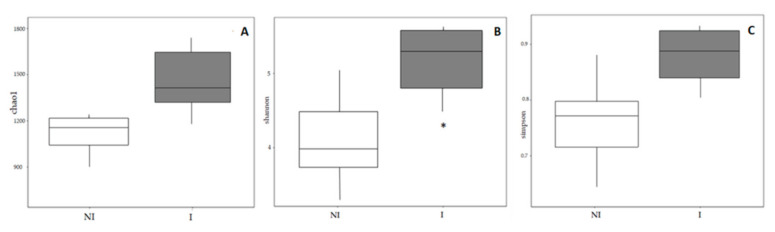
Alpha diversity metrics in the skin mucus of European sea bass. (**A**) Chao1 index (chao1), (**B**) Shannon–Wiener index (Shannon), (**C**) inverse-Simpson index (Simpson) in the mucus microbiota of non-infected (NI) and infected (I) European sea bass. Different indices are represented by Box-Whisker diagrams for the two experimental groups. Significant differences are indicated with an asterisk.

The diversity and richness of the bacterial populations from the skin mucus of two experimental groups were studied through alpha diversity metrics (Chao1, Shannon and inverse-Simpson indices). Total expected richness calculated by Chao1 index, as well as inverse Simpson diversity index, showed no significant differences between the two experimental groups. However, significant differences were observed in the Shannon diversity index (*p* < 0.05), it being significantly higher in the mucus of infected (I) fish compared to non-infected fish (NI) (Figure 7).

**Figure 8:**
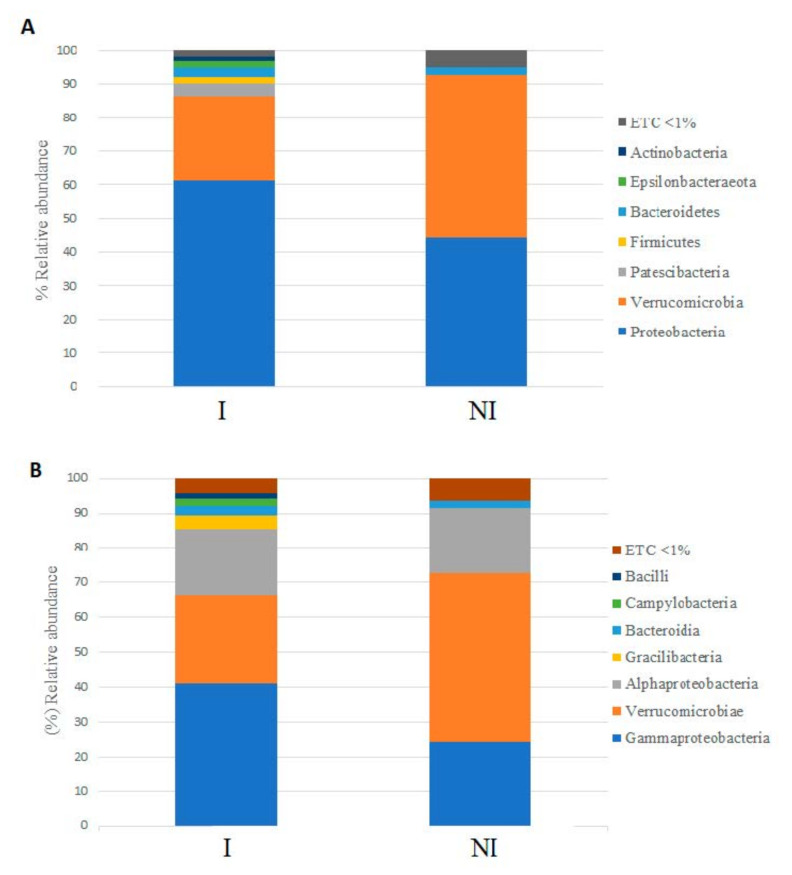
Relative abundance (%) of bacteria at the phylum (**A**), class (**B**), level in the skin mucus microbiota of non-infected (NI) and infected (I) fish. ETC: relative abundance < 1% in average.

Taxonomic composition at phylum level showed *Proteobacteria, Verrucomicrobia, Patescibacteria, Bacteriodetes, Firmicutes, Epsilonbacteraeota*, and *Actinobacteria* as the dominant phyla (relative abundance > 1%) (Figure 8A). When the mucus of non-infected fish was studied, *Verrucomicrobia* was the dominant phylum in the microbiota of non-infected fish (48.36%), followed by *Proteobacteria* (44.34%), *Bacteroidetes* (2.17%), *Actinobacteria* (0.91%), *Patescibacteria* (0.85%), *Firmicutes* (0.72%) and *Epsilonbacteraeota* (0.21%). On the contrary, *Proteobacteria* showed the highest relative abundance in the microbiota of infected fish (61.22%), followed by *Verrumicrobia* (25.20%), *Patescibacteria* (3.82%), *Bacteroidetes* (2.93%), *Epsilonbacteraeota* (1.90%), *Firmicutes* (1.88%) and *Actinobacteria* (1.18%).

At class level, 7 classes showed relative abundance percentages above 1% across samples: *Gammaproteobacteria*, *Verrucomicrobiae*, *Alphaproteobacteria*, *Gracilibacteria*, *Bacteroidia*, *Campylobacteria* and *Bacilli*. *Verrucomicrobiae* (48.36%) followed by *Gammaproteobacteria* (24.32%), *Alphaproteobacteria* (18.98%), *Bacteroidia* (2.15%), *Gracilibacteria* (0.79%), *Campylobacteria* (0.22%) and *Bacilli* (0.21%) were detected in the microbiota of non-infected fish. By contrast, higher *Gammaproteobacteria* abundance (40.97%) followed by *Verrucomicrobiae* (25.20%), *Alphaproteobacteria* (19.41%), *Gracilibacteria* (3.77%), *Bacteroidia* (2.88%), *Campylobacteria* (1.90%) and *Bacilli* (1.60%) were observed in the microbiota of infected fish. (Figure 8B)

**Figure 9:**
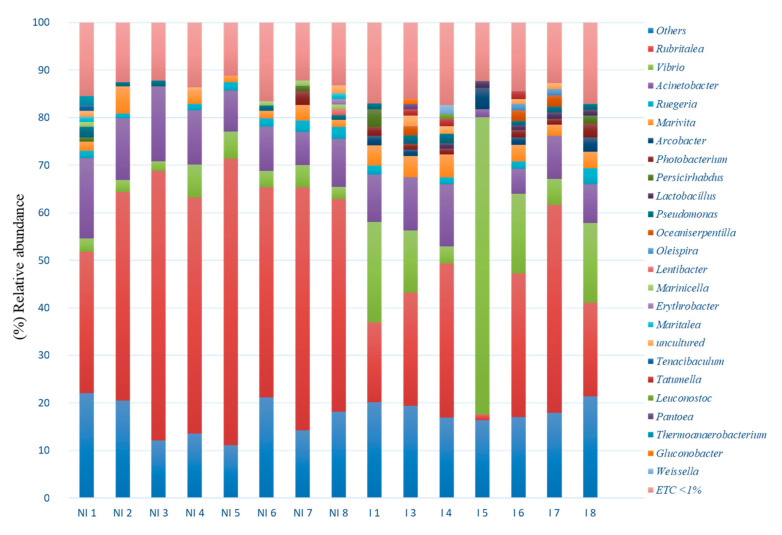
Relative abundance (%) at genus level of the individual skin mucus microbiota of non-infected (NI) and infected (I) fish. ETC: relative abundance < 1%.

When microbiota was considered at genus level, *Vibrio*, *Rubritalea*, *Acinetobacter*, *Marivita*, *Arcobacter*, *Persicirhabdus*, *Ruegeria*, *Photobacterium*, *Oceaniserpentilla* and *Pseudomonas* were detected with relative abundance percentages >1%. (Figure 9). Although with different percentages depending on the infection status, the most abundant genera corresponded to *Rubritalea* (47.47% and 23.96%, NI/I, respectively), *Acinetobacter* (11.48% and 8.35% NI/I, respectively) and *Vibrio* (3.84% and 19.92%, NI/I, respectively). However, the non-infected specimen (NI 1), showed abundance values of *Rubritalea* genus lower than in the case of other non-infected fish. In addition, genera showing abundance percentages below 1% represented about 30% of the total abundances, regardless of the health status of the specimens (Figure 9).

**Figure 10:**
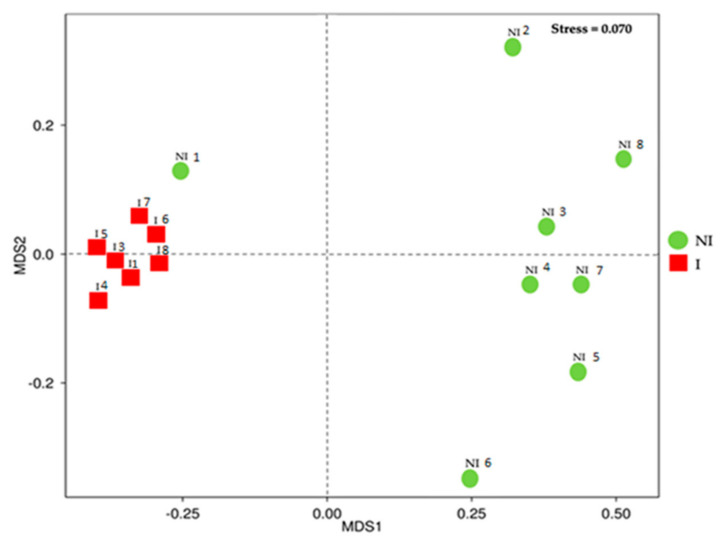
Non-metric Multi-dimensional Scaling (NMDS) in the skin mucus microbiota of non-infected (NI) and infected (I) European sea bass.

Beta diversity of non-infected and infected fish microbiota was determined and community structure studied by using non-metric multi-dimensional scaling (NMDS) (Figure 10). Mucus samples split out in two groups according to the OTUs detected in the mucus based on the infection status (Stress value 0.070). Analysis of similarity (Anosim) showed that there were significant differences across microbial communities between the two experimental groups (R-value 0.5215, *p* = 0.002). However, the microbiota pattern of one of the non-infected specimens (NI 1) was associated with the microbiota patterns of infected fish.

**Figure 11:**
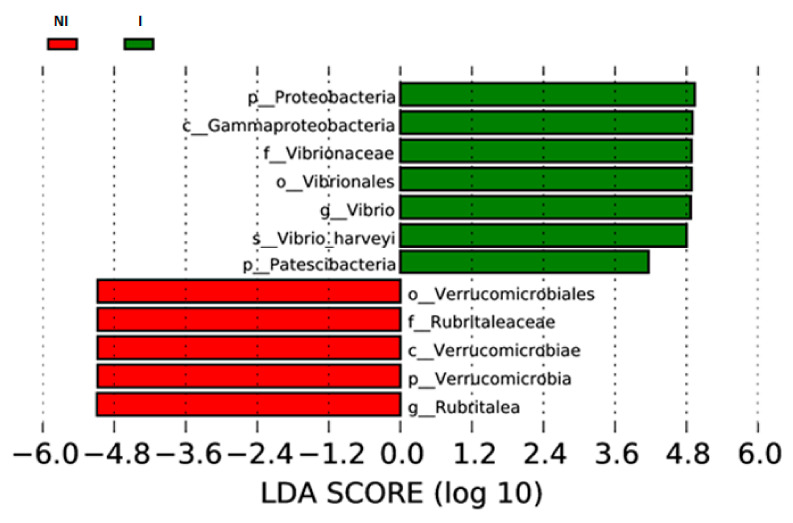
Linear discriminant analysis (LDA) effect size (LEfSe) analysis of skin mucus microbiota of European sea bass non-infected (NI) and infected (I) with *V. harveyi*. LDA scores for differentially abundant taxa in the microbiota of infected fish are shown in green and in the microbiota of non-infected fish in red. Prefixes represent abbreviations for taxonomic rank of each taxa, with phylum (p_), class (c_), order (o_), family (f_), genus (g_), and species (s_).

Finally, LEfSe was used to detect biomarkers. LEfSe analyses determined the taxa that most likely to explain differences between mucus from non-infected and infected fish. The species sorted by LDA score were mainly Proteobacteria (*V. harveyi*) for the infected group (I) while *Verrucomicrobia* (specifically the genus *Rubritalea*) was mainly associated with the non-infected group (NI) (Figure 11).

## 4. Discussion

Most infections caused by microorganisms (bacteria, virus or parasites) start at or affect the mucosal epithelia of fish [25]. Mucosal surfaces are exposed to a variety of antigens while coexisting with commensal and opportunistic microorganisms at the same time, collectively known as microbiota. [6]. In this study, *V. harveyi* was the identified causative agent of the skin ulcers observed in the animals. *V. harveyi* is not always recovered as a pure culture from diseased animals. Of course, it is uncertain whether the presence of two or more bacterial taxa from the same pathological material represents co-culture or the presence of secondary invaders or even chance contaminants [26]. One method for identifying changes in microbiota and pathways possibly involved in natural disease outbreaks, which is a main concern in the aquaculture industry, is to compare the microbiota of healthy and diseased fish from the same phylogeny, belonging to the same species, in the same developmental stage, and held under the same conditions (diet, rearing conditions, water quality, etc.) [27].

Vibriosis is among the most common diseases leading to massive mortalities in shrimp, fish and shellfish [3,28,29]. Several species of *Vibrionaceae* have been associated with diseases outbreaks in fish farms being the most common ones *V. parahaemolyticus*, *V. alginolyticus*, *V. harveyi*, *V. owensii* and *V. campbellii* [30]. The vibriosis infection may be transmitted through oral routes and external injuries might also play a crucial role in the transmission of the infection through the skin. It is also assumed that cohabitation between diseased fish and healthy ones can contribute to the disease dissemination. The incubation period is short (3 days) although it depends on many factors being crucial the virulence of the pathogen and the fish species susceptibility [4]. However, in spite of the importance of the mucosal surfaces in the process of infection and the prevalence of this disease in aquaculture, the immune response and the skin microbiome associated with this disease has not been studied yet. In fact, only a few skin microbiome studies comparing healthy and diseased fish have been published and, therefore, it is difficult to gain a comprehensive picture on how microbiota exerts effects on fish health status [31,32,33,34,35,36]. It is important to mention that none of these studies are related to a vibriosis outbreak. In the present study, the immune response at local and systemic levels and the skin mucus microbiome was studied in European sea bass after a natural disease outbreak associated to *V. harveyi*. The data obtained from infected and non-infected fish were studied and compared. As was explained in the methodology, in our facilities, the macroscopic skin lesions were observed in some animals from the different tanks. However, it is important to underline that finally, the infection was resolved alone without being administered any treatment to the fish. In other words, some fish remained always non-infected, in spite of being cohabiting with infected ones. More studies are needed to understand the different possible mechanisms of infection developed by this important marine fish pathogen. Fish mucus and skin/scales serve as a natural barrier to pathogens and foreign substances, containing glycoproteins or mucopolysaccharide proteins produced mainly by goblet cells [37,38]. The mucus also contains innate humoral molecules and specific antibodies. In this study, several parameters of the immune system were studied in order to establish the main mechanisms involved in the immune response against a natural outbreak of vibriosis with the aim to develop correct measures to reduce the incidence of such disease [7]. Regarding the mucosal immune response in skin (local response taking into account the injuries caused in this organ by the presence of the pathogenic bacteria), a significant decrease in protease activity was observed in the skin mucus of fish infected by *V. harveyi*. These enzymes may work directly on bacterial pathogens, cleaving their proteins and causing the death of the bacteria [8]. Proteases also prevent pathogen invasion by altering mucous consistency and lead to an increase in sloughing of mucus. It has also been suggested that the reduction of protease by several stressors in fish skin mucus is related to defence against bacterial or parasite infections [39]. Besides this, proteases can also enhance the production of other innate immune components such as complement, immunoglobulins (Igs) or antibacterial peptides [7]. However, in the present study, no significant differences were observed in Igs (total Igs or IgM) between both groups of fish (infected and non-infected), probably due to the fact that it was still too early in the infection process (only a few days) to see some effect in the adaptive immunity caused by the pathogen. These results might indicate the importance of protease activity in the local immune response and the possibility of using this activity as a disease biomarker.

Concerning the systemic immune response, again a significant decrease in protease activity in serum of fish infected by *V. harveyi* was also reported, which could be related to the local immune response. Moreover, lysozyme activity was significantly lower in serum of fish infected in comparison to non-infected fish. Lysozyme is an important defense molecule of the innate immune system, which is significant in mediating protection against microbial invasion [40]. The reduction of these two activities (proteases and lysozyme) in fish serum might indicate again that the immune system might be using such defense mechanisms to overcome the disease. By contrast, an increase was observed in the values of peroxidase activity in serum of infected fish. The present results agree with those observed in Senegalese sole (*Solea senegalensis*) after 7 days of a bath challenge with *Tenacibaculum maritimum* [39]. In the case of peroxidases, these enzymes effectively eliminate H_2_O_2_ and maintain the redox balance of the immune system [13] and also of the other organs and system of organisms. The fact that this activity is significantly higher in infected fish from this study could indicate that the fish were fighting against the infection by using the humoral innate immune instruments. Moreover, the bacteria involved in the infection are probably inducing the production of a reasonable amount of reactive oxygen species (ROS) and serum peroxidase activity reduces such an imbalance [41]. Moreover, it is tempting to consider that such enzyme is essential for immunity against bacteria and that other enzymes important in redox balance such as glutathione peroxidase, catalase, and superoxide dismutase should be studied in future studies to understand the role and implications of ROS in fish health and disease. In the present study, it was not possible due to the low serum volume that was obtained from each sampled specimen because of fish size.

Species diversity and richness have an effect on the role and stability of microbial ecosystems [42,43]. Regarding microbial richness and diversity in diseased fish, different outcomes have been reported, indicating that infection does not always result in losses of overall microbial diversity. A study demonstrated that stress by transportation resulted in an increased number of culturable skin mucus bacteria in rainbow trout [44]. In a recent analysis using laboratory seawater Atlantic salmon (*Salmo salar*), the skin microbiome diversity of control and salmon alphavirus-infected fish, showed no major differences between them. However, experimentally infected salmon lost the majority of the Proteobacteria and had increased abundances of opportunistic taxa (*Flavobacteriaceae, Streptococcaceae, Tenacibaculum*) [34]. In another study, the viable skin mucus microbiome of Atlantic salmon and smallmouth bass (*Micropterus dolomieu*) was studied using a plate count technique. Bacterial diversity was evaluated over time following natural *Aeromonas salmonicida* outbreaks in a fish farm [45]. The authors concluded that microbial diversity decreased over time due to an overrepresentation of *A. salmonicida* in the bacterial community. Other conditions such as gut enteritis have been associated with decreased skin microbiota diversity in *Seriola lalandi* [27]. On the same note, another study indicated that Atlantic salmon experiences a loss of microbial richness, but an increase in diversity while a destabilization of the skin microbiota composition happened after an infection by salmon lice (*Lepeophtheirus salmonis*) [31]. Curiously, an ich (*Ichthyophthirius multifillis*) infection in rainbow trout also led to a rapid increase in diversity and an overall shift in the skin microbiota [36]. In the present study, when comparing alpha-diversity, results showed that fish infected with *V. harveyi* showed a significantly higher diversity (Shannon index) in the microbial community in comparison to the non- infected group. Complementary results to those obtained in the Chao1 and inverse Simpson index, in which there was an increase in diversity in the infected group, although these differences were not reported to be statistically significant. The indices indicated that infected mucus skin samples contained more diverse bacterial communities compared to non-infected samples. In addition, significant changes in beta-diversity occurred in the skin mucus of European sea bass, indicating that the microbial communities from infected and not infected fish were significantly different, showing clear signs of dysbiosis and a shift in the overall microbiota composition. In the NMDS analysis, the non-infected sample clustered with the infected group (NI 1) also corresponded to an asymptomatic fish at the time of sampling. This sample could represent an early stage of the infection; however, there was no opportunity to follow the evolution of the infection and determine if the infection symptoms finally would have an outcome. The methodology used to categorize fish as infected or non-infected is based on the recovery of bacterial cells capable to grow on bacteriological media. Factors such as the pathogen concentration or viability on in vitro media determine its recovery on bacteriological media. However, they do not imply that the microorganism is not able to cause the infection in the host.

Host microbiota play an important role in the control of pathogens, it being able to prevent and control infection by mechanisms such as niche exclusion, competition for nutrients or antagonism [46]. Skin microbiota of gilthead sea bream infected by *V. harveyi* presented modified abundance percentages of different bacterial taxa. Altered microbial communities may imply a loss of the ability to control pathogen populations. In this way, another recent study observed that a combination of culturable bacterial species from rainbow trout (*Oncorhynchus mykiss*) microbiota was able to confer protection against *Flavobacterium columnnare* infections, inhibiting pathogen population [47]. Similarly, resistance to *F. columnare* provided by a core microbiota that conferred a community-level protection has also been demonstrated [48].

In addition, a role in the modulation of the immune response against pathogens has been attributed to host microbiota [46]. Thus, commensal bacteria are able to induce inflammatory response [49] and activate innate immune response [50], all essential for the fight against pathogens in fish. Whether identified taxa in the microbiota of infected gilthead sea bream is responsible for decreased peroxidase and lysozyme activities observed in the present study needs to be determined in further studies.

The skin microbiota of European sea bass infected with *V. harveyi* was dominated by the Proteobacteria phylum followed by *Verrucomicrobia* and *Patescibacteria* phyla; meanwhile, in the non-infected group the dominant phylum was found to be *Verrucomicrobia* followed by *Proteobacteria* and *Firmicutes*. In contrast to our results, researchers studying the microbiota of different skin areas of seabass observed that biodiversity of microbial communities was different depending of the skin area studied, but in none of them was the *Verrucomicrobia* phylum one of the most predominant phyla reported. It has been reported that changes in the phylogenetic diversity of skin microbiota can produce alterations of its functionality to induce disease [51], and in agreement with this conclusion, in our study the comparison between skin microbiota of infected and non-infected specimens showed that the phylogenetic diversity was affected quantitatively. In this way, the abundance of *Proteobacteria* was significantly higher in the infected group in comparison to the non-infected group, while *Verrucomicrobia* abundance was significantly higher in the non-infected group. These differences could be due the fact that *V. harveyi*, belonging to the *Proteobacteria* phylum, was the most probable causative agent of the skin ulcers. *Proteobacteria* and *Bacteroidetes* have been reported as the predominant phyla in the skin microbiota of different fish species [52,53]. In particular, in a recent study which aimed to establish a baseline microbiome in European sea bass, *Proteobacteria* and *Bacteroidetes* were reported as the most abundant phyla [53,54,55]. However, in this study, Bacteroidetes represented less than 3% in both groups. Several factors known to impact microbiome composition, such as age, seasonality or water temperature, could be further driving such differences. With respect to the class, *γ-Proteobacteria* was found to be the predominant class of the infected group (41% approximately) and *Verrucomicrobiae* was the dominant class of the non-infected group (48% approximately). Significant differences were observed at the class level, γ-*Proteobacteria*, were significantly more abundant in the infected group while *Verrucomicrobiae* was highly representative for the not infected group. The high prevalence of γ-*Proteobacteria* in the infected group might be due to the fact that *V. harveyi* is indeed the causative agent of the infection.

*Rubritalea* was the dominant genus in the mucus microbiota of non-infected fish, it being a biomarker of this group. The presence of *Rubritalea* has been reported in the mucus, both skin and gills, of healthy European seabass [54,55]. Several studies have described that this bacterial genus had the capacity to produce carotenoids and squalene [56,57]. Squalene is a linear triterpene precursor for the synthesis of secondary metabolites such as sterols, hormones, or vitamins [56] and it has been reported with antioxidant and antimicrobial activity [57]. Furthermore, carotenoids act as antioxidants and are precursors of vitamin A. For this reason, the presence of *Rubritalea* in asymptomatic fish could be associated with a healthy state of the skin barrier. It could be related with the event that the NMDS analysis showed that the microbiota pattern of one (NI 1) of the non-infected specimens was associated with the microbiota patterns of infected fish. In this specimen the abundance of *Rubritalea* was lower compared to the abundance of this genus in the other non-infected specimens. On the other hand, in this fish the abundance of *Vibrio* was not as elevated as in infected specimens. These results suggest the modulation of the skin microbiota of this fish towards a microbiota pattern more similar to the infected fish but without symptoms due to the lower abundance of *Vibrio*.

Thus, we can suggest that in this case, *Vibrio* is the key taxa associated with diseased fish. In similar studies in which the microbial communities in skin ulcers were studied, *Tenacibaculum* and *Arcobacter* species were found to be dominating the cutaneous skin and ulcer mucus of Atlantic salmon [31]. *Vibrio*, *Tenacibaculum*, *Flavobacterium* and *Pseudomonas* have also been associated with skin diseases of Atlantic salmon [34,58]. Therefore, our results demonstrated that European sea bass infected with *V. harveyi* suffered a microbial composition shift in the skin mucus.

Proteobacteria was the dominant phyla colonizing the skin mucus and out-competed other phylum, especially *Verrucomicrobia*, in agreement with other studies [34,35,36,59]. Moreover, the infection caused by *V. harveyi* was associated to changes in the bacterial composition and also resulted in differences in the host mucosal immune response. Peroxidase seemed to have an important role in the course of vibriosis infection. Meanwhile, protease and lysozyme seemed to have been already exhausted.

To conclude, characterizing the mucosal surfaces and microbial composition of the economically important fish species, such as the European seabass, is of vital importance in order to detect potential disease outbreaks in fish farms. In the near future, metagenomic and transcriptomic studies would be helpful in understanding the functionality of fish microbiomes and their interaction with the fish immune system. In fact, manipulating the microbiota in aquaculture may become a powerful way to improve water quality, inhibit the growth of pathogens, and boost the immune response of fish. Identified biomarkers of the immune responses and microbiota detected in the present study can contribute to the early-detection system of this disease in aquaculture and avoid significant losses. Further studies with a larger sample size should be undertaken to corroborate these results.

## Figures and Tables

**Table 1:** Bacterial strains isolated from European sea bass skin lesions and internal organs. MK, mid-kidney and HK, head-kidney.

Bacterial Characterization (16S Ribosomal RNA Gene)		
Location Site	Bacterial Strain	Partial Sequence (%)
Skin	*Vibrio harveyi* strain 2SYX001	97.48
Skin	*Vibrio harveyi* ATCC:35084	95.88
Skin	*Vibrio harveyi* strain SETBT4	96.88
Skin	*Vibrio harveyi* strain DS1810-S6_1	97.23
Skin	*Vibrio harveyi* strain SF-1	96.33
Skin	*Vibrio harveyi* strain NBRC	97.25
Skin	*Pseudoalteromonas* sp. strain 4634	97.40
Skin	*Pseudoalteromonas* sp. QD254Down-1	96.46
Skin	*Pseudoalteromonas* sp. QD254Down-1	98.07
MK	*Vibrio harveyi* strain HW	96.85
MK	*Vibrio harveyi* strain HW	96.92
MK	*Vibrio harveyi* strain HW	96.91
HK	*Vibrio harveyi* strain SF-1	97.17
HK	*Vibrio harveyi* strain HW	96.23
HK	*Pseudoalteromonas lipolytica* strain M4C_0m_07	97.94

## Data Availability

The data that support the findings of this study will be available on request from the corresponding author.
